# Characterization of chromosomal and megaplasmid partitioning loci in *Thermus thermophilus* HB27

**DOI:** 10.1186/s12864-015-1523-3

**Published:** 2015-04-18

**Authors:** Haijuan Li, Angel Angelov, Vu Thuy Trang Pham, Benedikt Leis, Wolfgang Liebl

**Affiliations:** Lehrstuhl für Mikrobiologie, Technische Universität München, Emil-Ramann-Straße 4, D-85354 Freising-Weihenstephan, Germany

**Keywords:** Partitioning genes (*par*), *Thermus thermophilus*, Chromosome, Megaplasmid, ParB

## Abstract

**Background:**

In low-copy-number plasmids, the partitioning loci (*par*) act to ensure proper plasmid segregation and copy number maintenance in the daughter cells. In many bacterial species, *par* gene homologues are encoded on the chromosome, but their function is much less understood. In the two-replicon, polyploid genome of the hyperthermophilic bacterium *Thermus thermophilus*, both the chromosome and the megaplasmid encode *par* gene homologues (*parABc* and *parABm*, respectively). The mode of partitioning of the two replicons and the role of the two Par systems in the replication, segregation and maintenance of the genome copies are completely unknown in this organism.

**Results:**

We generated a series of chromosomal and megaplasmid *par* mutants and sGFP reporter strains and analyzed them with respect to DNA segregation defects, genome copy number and replication origin localization. We show that the two ParB proteins specifically bind their cognate centromere-like sequences *parS*, and that both ParB-*parS* complexes localize at the cell poles. Deletion of the chromosomal *parAB* genes did not apparently affect the cell growth, the frequency of cells with aberrant nucleoids, or the chromosome and megaplasmid replication. In contrast, deletion of the megaplasmid *parAB* operon or of the *parB* gene was not possible, indicating essentiality of the megaplasmid-encoded Par system. A mutant expressing lower amounts of ParABm showed growth defects, a high frequency of cells with irregular nucleoids and a loss of a large portion of the megaplasmid. The truncated megaplasmid could not be partitioned appropriately, as interlinked megaplasmid molecules (catenenes) could be detected, and the ParBm-*parSm* complexes in this mutant lost their polar localization.

**Conclusions:**

We show that in *T. thermophilus* the chromosomal *par* locus is not required for either the chromosomal or megaplasmid bulk DNA replication and segregation. In contrast, the megaplasmid Par system of *T. thermophilus* is needed for the proper replication and segregation of the megaplasmid, and is essential for its maintenance. The two Par sets in *T. thermophilus* appear to function in a replicon-specific manner. To our knowledge, this is the first analysis of Par systems in a polyploid bacterium.

**Electronic supplementary material:**

The online version of this article (doi:10.1186/s12864-015-1523-3) contains supplementary material, which is available to authorized users.

## Background

All living cells have mechanisms ensuring the faithful segregation of the replicated genomes to the daughter cells. While the tubulin-based mitotic apparatus for DNA segregation used by eukaryotes is well studied, the mechanisms that mediate chromosome segregation in prokaryotic cells are less well understood. Evidence from several groups demonstrates that bacterial chromosomes are also actively segregated and this segregation does not rely on cell growth [1-4]. Also, it has been shown that cytoskeletal proteins are also present in prokaryotic cells and they form mitotic-like apparatuses that provide force for active chromosome segregation [5,6].

Several elements have been proposed which may make contributions to the dynamic movement of bacterial chromosomes [5,7]. For example, it has been suggested that DNA polymerase can provide force for bidirectional chromosome segregation in *Bacillus subtilis* cells [8,9]. Likewise, RNA polymerase has also been implicated to afford both incentive force and directionality for segregation through interacting with origin-proximal regions [10,11]. MreB is a chromosomally encoded actin homolog and in some rod-shaped bacteria it has been shown that MreB not only determines the cell shape, but is also involved in chromosome segregation [12-14].

Partitioning (*par*) genes have been known for a long time to play a pivotal role in the maintenance of low-copy-number plasmids. Plasmid *par* locus usually contain three components: two ORFs encoding an ATPase (ParA) and a DNA-binding protein (ParB), and a centromere-like specific DNA sequence (*parS*). ParB binds its corresponding *parS* sequence, forming a large nucleoprotein complex. Low-copy-number plasmids with disrupted *par* loci localize improperly and are thus readily eliminated from host cells [15,16]. The molecular mechanisms by which *par* loci segregate plasmids have been studied to some extent. It has been suggested that ParA can form filaments which interact with ParB-*parS* complexes and provide force for segregation [17-20].

Many bacterial chromosomes encode orthologs of the plasmid partitioning proteins (Par) near their origin regions [21]. The first ParB-binding chromosomal *parS* sites were discovered in *B. subtilis*, where 10 pseudopalindromic 16-bp sequences were identified in the 20% origin-proximal region of its chromosome. The presence of merely one such site could prevent the loss of an otherwise unstable plasmid from the host cell in a ParAB-dependent manner [22,23]. The consensus for the 16-bp sequence is 5′-TGTTNCACGTGAAACA-3′. Recently, this 16-bp sequence has been found in a large variety of bacteria, and in most cases, these sequences are origin-proximally located [24,25]. Normally, the corresponding *parAB* genes can also be identified in such *parS*-containing chromosomes. While the crucial role of the *par* loci in plasmid partitioning has been well studied, their chromosomal counterparts are relatively poor investigated and the role of these Par systems in chromosome segregation is still disputable. The available data [26] support a model called diffusion ratchet mechanism where ParA uses the nucleoid as a matrix and *ori*-*parS*-ParB foci move by following a retracting ParA cloud.

Mutations introduced in chromosomal *par* loci usually have pleiotropic effects. In *B. subtilis* the chromosomal *parAB* orthologs are not essential genes, but they are involved in chromosome replication and segregation, chromosome origin localization and separation, and developmental gene regulation [27-29]. The *parAB* genes in *Caulobacter crescentus* on the other hand are essential and their depletion or overexpression results in defects in cell-cycle progression, cell division and chromosome segregation [30,31]. Each of the two chromosomes (chrI and chrII) of *Vibrio cholerae* contains a *par* locus (*parABS1* and *parABS2*). It has been shown that *parABS1* is probably involved in the segregation of the origin regions of chrI, but not of the bulk DNA of chrI or chrII [32,33]. In contrast, *parABS2* can promote accurate subcellular localization and maintenance of the bulk DNA of chrII but not of chrI [34].

Although these diverse functions of chromosomal Par systems have been revealed to some extent in a few model organisms, the situation in other bacteria remains largely unknown, especially in bacteria containing more than one replicon. In addition to *V. cholerae*, there is only one study related to the *par* loci in bacteria possessing multiple replicons. *Burkholderia cenocepacia* has three chromosomes and a low-copy-number plasmid. Dubarry and co-workers [35] identified *parS* sites on the four replicons, and showed that the respective *parABS* systems are independent of each other.

*Thermus thermophilus*, which belongs to the phylogenetically deeply branching *Deinococcus-Thermus* phylum, has been established as a model organism for studying thermophilic bacteria. The genome of *T. thermophilus* consists of a chromosome (1.89 Mb) and a megaplasmid (0.23 Mb). It has been recently shown that *T. thermophilus* strains are polyploid and the chromosomal and megaplasmid copy number of the HB8 strain has been estimated to be four or five [36]. There are no reports in the literature regarding the chromosome and megaplasmid segregation in this organism, and the chromosome segregation mechanisms in polyploid bacteria have only recently begun to be addressed, e.g. in some polyploid cyanobacterial species [37,38]. In *T. thermophilus*, *parAB* gene homologues (termed here *parABc* and *parABm*, respectively) are also present both on the chromosome and on the megaplasmid [39]. Two studies have investigated the biochemical and structural properties of the chromosomal ParAc and ParBc proteins of *T. thermophilus* [40,41]. In this work, we address the functions of the chromosomal and megaplasmid *par* loci in *T. thermophilus*. We performed *in vitro* DNA binding assays with heterologously expressed ParB proteins, and generated a series of chromosomal and megaplasmid *par* mutants and sGFP reporter strains for subsequent analysis with respect to growth and DNA segregation defects, genome copy number and replication origin localization. The results from these experiments give first insights into how the two Par systems function in parallel in this thermophilic and polyploid bacterium.

## Results

### Genetic organization of the *par* loci in *T. thermophilus*

**Figure 1:**
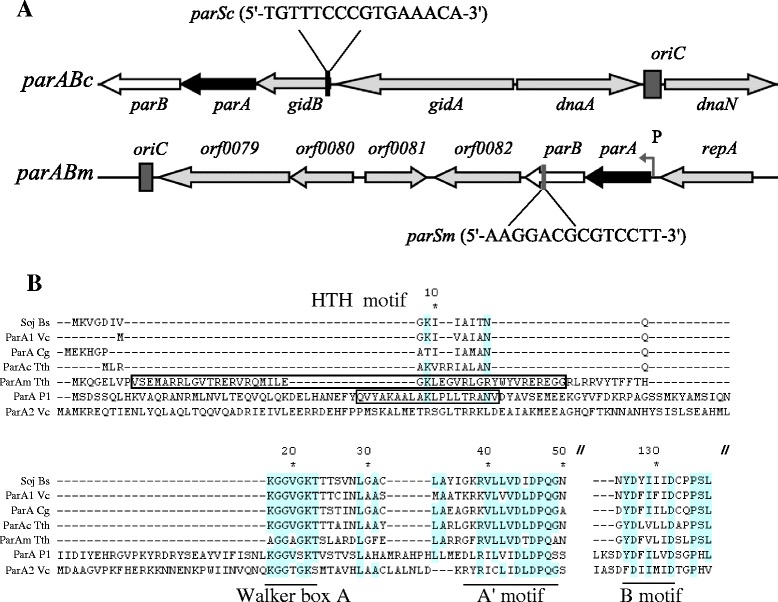
Organization of the chromosomal and megaplasmid *par* loci, and features of the corresponding ParA proteins. **(A)** Schematic organization of the *parABc* and *parABm* regions. Genes encoding proteins with unknown functions are labeled with their ORF numbers. The predicted replication origins in the chromosome and megaplasmid are indicated with dark-gray bars. The positions and sequences of the chromosomal and megaplasmid *parS* sites are shown. **(B)** Part of a multiple sequence alignment of *T. thermophilus* ParAc, ParAm and ParA proteins from representative bacterial species. Abbreviations: Bs, *B. subtilis*; Vc, *V. cholerae*; Cg, *Corynebacterium glutamicum*; Tth, *T. thermophilus*; P1, *E. coli* P1 phage. Completely conserved amino acids are shown with blue color. Black frames indicate the predicted helix-turn-helix (HTH) motifs identified in P1 ParA, and Tth ParAm.

Both the chromosome and megaplasmid of *T. thermophilus* contain *par* loci [39], termed here *parABc* and *parABm*, respectively. The chromosomally encoded *par* locus consists of *parAc* (TT_C1605), *parBc* (TT_C1604) and *parSc*, organized in a way often found in bacterial chromosomes (Figure 1A). According to one report [42] and our own GC skew analysis (Additional file 1: Figure S1A), the chromosome replication origin (*oriC*) is positioned downstream of *dnaA* (TT_C1608), thus the *parABc* locus is located close to *oriC* (~6 kbp distance) (Figure 1A). The *parSc* site was identified using the 16-bp consensus sequence (5′-TGTTNCACGTGAAACA-3′) allowing for one base pair mismatching and only one site was found [24]. When two or three mismatches were allowed, no other sequences could be found. The *parSc* sequence is located within a gene (*gidB*) encoding 16S rRNA methyltransferase immediately upstream of *parAc* (Figure 1A). Based on GC skew analysis, the cumulative minimum indicating the megaplasmid replication origin position was observed around the open reading frame of TT_P0079 (Additional file 1: Figure S1B; Figure 1A), thus the megaplasmid *parAB* genes (TT_P0084 and TT_P0083) are also in the vicinity of the predicted megaplasmid *oriC* (Figure 1A). One 14-bp palindromic sequence (5′-AAGGACGCGTCCTT-3′) was found in the *parBm* gene, and we provide evidence that this sequence could serve as the megaplasmid *parS* site (i.e. ParBm binding site, see below).

The organization of the *T. thermophilus* chromosomal *par* locus is similar to that of other bacterial chromosomal *par* systems [42]. The structure and genetic context of the *parABm* operon, on the other hand, is similar to the situation in low-copy-number plasmids where the *par* genes are adjacent to the *repA* gene for a plasmid-like replication initiator and are flanked by direct repeats. The chromosomal and megaplasmid Par proteins also possess different features. While both ParAc and ParAm are Walker-type ATPases which contain a conserved P-loop ATP binding motif, they differ in their sizes (249 aa for ParAc and 322 aa for ParAm) as well as in the presence of an N-terminal helix-turn-helix motif (HTH) in ParAm (Figure 1B), a feature that normally appears in plasmid ParAs but not in their chromosomal counterparts [21].

### Generation of *par* mutants in *T. thermophilus*

**Figure 2:**
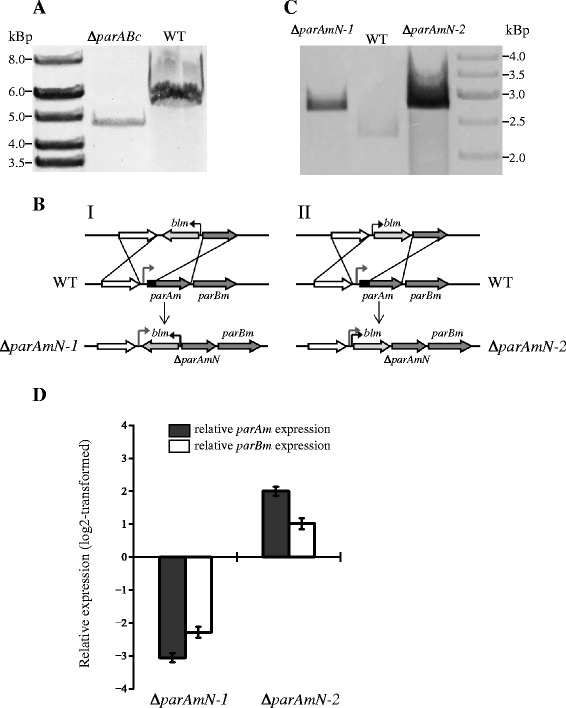
Generation and genotype confirmation of the chromosomal and megaplasmid *par* mutants. **(A)** Genotype confirmation of the *parABc* mutant by Southern blot. The genomic DNA was digested with BamHI and hybridization was performed with a 992-bp biotin-labeled DNA fragment. The *in silico* predicted sizes are 5.13 kbp for the wild type and 4.68 kbp for Δ*parABc.* **(B)** Schematic diagrams showing exchange of the N-terminus-encoding region of ParAm (amino acid positions 1–40) with *blm*. Gray arrowhead denotes the promoter region of *parABm*; black arrowhead, promoter of *blm*. **(C)** Genotype confirmation of the Δ*parAmN-1* and Δ*parAmN-2* mutants by Southern blot. The genomic DNA was digested with PstI, the predicted sizes are 2.26 kbp for the wild type and 2.73 kbp for Δ*parAmN-1* and Δ*parAmN-2*. **(D)** Transcription levels of the truncated *parAm* and *parBm* genes in Δ*parAmN-1* and Δ*parAmN-2* relative to those of the wild type determined by RT-qPCR. Gray bar represents the relative expression level of the truncated *parAm*, white bar represents that of *parBm*. The average values and SDs shown are from three experiments.

We initiated the investigation of the two distinct *par* loci in *T. thermophilus* by attempting to generate deletion mutants. The chromosomal *parAB* genes were replaced by a thermostable kanamycin resistance gene cassette (*kat*). Southern blot analysis showed complete deletion of the *parABc* operon in the resulting mutant, Δ*parABc* (Figure 2A). In the same way, we initially tried to replace the whole *parABm* operon in the megaplasmid with a thermostable bleomycin resistance gene (*blm*). However, we were unable to obtain a null mutant: all the resulting transformants were found to be heterozygous, containing both the wild-type and the mutant alleles at the target locus (Additional file 2: Figure S2A). It is important to note that *T. thermophilus* is polyploid, and thus the heterozygous state is possible [36]. Thus, there appeared to be a strong selective pressure to retain *parABm*, i.e. *parABm* is probably essential. We then tried to knock down the *parABm* operon in order to test whether a perturbation in the amounts of ParAm and/or ParBm would yield a detectable phenotype, and also to pinpoint which of the two ORFs is essential. We constructed partial deletion variants of the *parAm* gene lacking the first 40 codons at the 5′-end of the coding sequence but possessing the ribosome-binding sequence and start codon. Upstream of the new ORF start, the variants carried the *blm* marker with its strong promoter either in the opposite (Δ*parAmN-1*) or in the co-linear orientation (Δ*parAmN-*2) relative to the direction of *parABm* transcription (Figure 2B). Further, we also attempted to delete *parBm* by exchanging it with *blm*. Complete exchange of *parBm* was not possible (Additional file 2: Figure S2B), while deletion of the N-terminus-encoding sequences of the *parAm* was successful. Both anticipated deletion variants, which differed only in the orientation of the *blm* marker upstream of the truncated *parAm* ORF, were obtained as judged by Southern blot analysis (Figure 2C). We then determined the transcription levels of the truncated *parAm* and *parBm* genes in the Δ*parAmN-1* and Δ*parAmN-2* strains by RT-qPCR. As expected, in Δ*parAmN-1* the transcription levels of both genes were decreased: the relative mRNA amounts were 10-fold (for the truncated *parAm*) and 5-fold (for *parBm*) lower than that in the wild type, while in Δ*parAmN-2* the levels of transcription of the two genes were 4-fold (for the truncated *parAm*) and 2-fold (for *parBm*) higher than that of the wild-type strain (Figure 2D).

### Growth rate and frequency of cells with aberrant nucleoids in the *par* mutants

**Figure 3:**
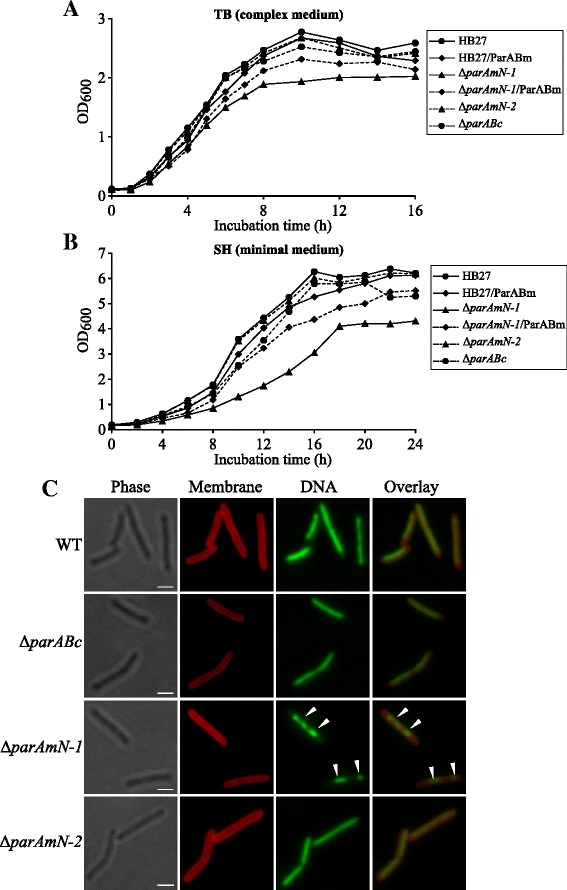
Growth phenotypes, cell shape and nucleoid morphology observations of the *par* mutant strains. **(A and B)** The cultures of the mutants were grown in antibiotic-free complex medium (TB) **(A)** and minimal medium (SH) **(B)**. For complementation experiments, the wild type and the Δ*parAmN-1* strains carrying the plasmid pMK-*parABm* were grown in the presence of kanamycin (20 μg/ml). One representative of three independent experiments is shown. **(C)** Microscopic analysis of the cell shape, cell division and DNA morphology of the wild type and *par* mutants grown in complex medium (TB). Shown are representative phase-contrast (Phase) and fluorescence images (Membrane, DNA) and a merge between the membrane and DNA images (Overlay). The cells were stained with carboxyfluorescein (for membranes) and with DAPI (for DNA) before imaging. White arrows show aberrant nucleoids. Scale bars, 2 μm.

We analyzed the growth rates in complex (TB) and minimal (SH) media of the *par* mutants. In both media, the Δ*parABc* strain did not display a considerable growth defect (Figure 3A and B). The Δ*parAmN-1* mutant grew more slowly and reached a lower final optical density in both media tested, while in Δ*parAmN-2*, which differs from Δ*parAmN-1* only by the direction of the resistance marker, these growth defects were not observed (Figure 3A and B). The cell growth defect of Δ*parAmN-1* could also largely be complemented by introducing pMK-*parABm* and thereby providing a plasmid-borne copy of *parABm* (Figure 3A and B). Next, we performed microscopy experiments in order to examine cell morphology, DNA segregation and cell division of the Δ*parABc*, Δ*parAmN-1* and Δ*parAmN-2* mutants. When grown in complex medium, no cell morphology or cell division defects occurred in any of the mutants (Figure 3C). The frequencies of cells with aberrant nucleoids (as judged by the size and form of the DAPI-stained area) of Δ*parABc* grown in either TB or SH medium were indistinguishable from those of the wild type (Table 1). Together with the growth phenotype (Figure 3A and B), these data suggested that deletion of *parABc* did not lead to appreciable changes in the genome bulk nucleoid replication or segregation. In the case of the Δ*parAmN-2* strain, the frequency of cells with irregular nucleoids was also not affected (Table 1), while in strain Δ*parAmN-1* an increased number of cells were found to contain apparently less and patchy-staining nucleic acid when grown in TB medium (Figure 3C; Table 1). These experiments showed that inadequate amounts of the megaplasmid ParAB proteins lead to genome segregation and/or replication defects and provoke defective cell growth.

**Table 1:** **Frequencies of cells with aberrant nucleoids and relative genome copy numbers in** ***par*** **mutants and ParAm/ParBm overexpression strains**

**Strain**	**Cells with aberrant nucleoids (%)**	**Relative TT_P0043 copies**	**Relative** ***term*** **copies**	**Relative** ***oriCc*** **copies**	**Relative** ***terc*** **copies**
WT	1.24	1	1	1	1
Δ*parABc*	3.05	1.13 ± 0.04	1.09 ± 0.05	1.13 ± 0.15	1.14 ± 0.08
Δ*parAmN-1*	33.02	/	1.52 ± 0.05	0.91 ± 0.13	0.93 ± 0.23
Δ*parAmN-2*	2.28	1.24 ± 0.09	1.26 ± 0.16	1.08 ± 0.07	1.12 ± 0.02
TMP01	1.26	3.06 ± 0.23	2.45 ± 0.19	0.93 ± 0.17	0.85 ± 0.12
TMP02	2.12	2.84 ± 0.14	2.29 ± 0.35	0.92 ± 0.09	0.89 ± 0.07
TMP0	1.15	0.84 ± 0.26	0.95 ± 0.05	0.97 ± 0.07	1.05 ± 0.03

The frequencies of cells with aberrant nucleoids were measured in cultures grown in TB medium; approximately 300 cells were analyzed for each strain. Relative genome copy numbers were determined by quantitative PCR. The mean values and the standard deviations of three independent experiments are shown. “/” indicates undetectable.

### Genome content analysis of the *par* mutants

**Figure 4:**
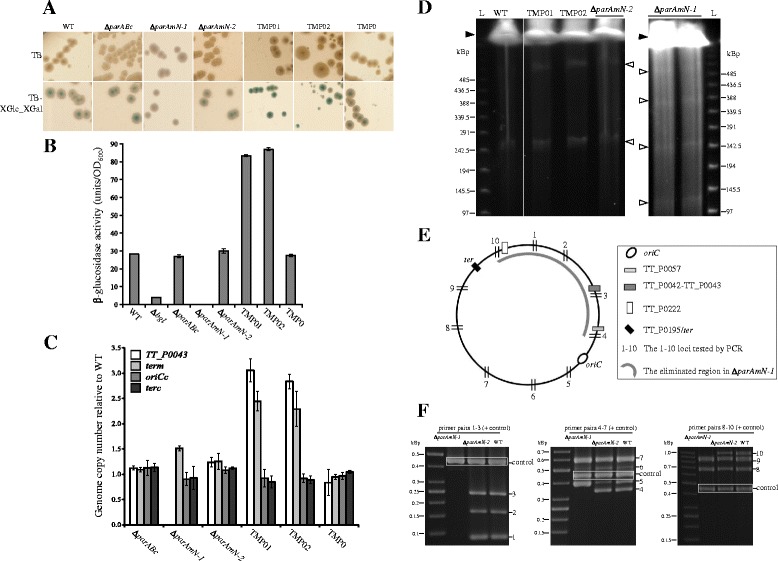
Characterization of genome features of the chromosomal and megaplasmid *par* mutants and ParAm/ParBm overexpression strains. **(A)** Phenotypes of the strains on complex media (TB) and on TB supplemented with the chromogenic substrates XGlc and XGal. **(B)** Intracellular β-glucosidase activity measurements of the strains. The Δ*bgl* strain was used as a negative control. The means and the SDs of three independent experiments are shown. **(C)** Relative chromosome and megaplasmid copy numbers of the individual mutants determined by qPCR. The means and SDs are from three experiments. **(D)** Pulsed field gel electrophoresis visualizing chromosome and megaplasmid. “L”, lambda ladder; the positions of the chromosome and megaplasmid are indicated with black and white arrows. **(E)** Schematic drawing of the megaplasmid pTT27. The positions of the primer pairs used for detecting the megaplasmid sequence loss in Δ*parAmN-1* are indicated with short black lines and numbers from 1 to 10. The loci on the megaplasmid that have been investigated are indicated with different bars, and their names are on the right panel of the figure. **(F)** PCR amplification results for the 10 loci indicated in **(E)** from wild type, Δ*parAmN-1* and Δ*parAmN-2*. The primer pairs 1 to 3, 4 to 7 and 8 to 10 were mixed into three pools, and in each reaction amplification of a chromosomal gene locus (*pyrF*) was used as a control. The predicted sizes of the PCR products 1 to 10 are 87, 164, 247, 346, 400, 498, 610, 699, 898 and 1014 bp. The size of the control amplicon is 460 bp (white frame). The bands of the 10 PCR products are indicated with numbers 1–10 on the right side of the corresponding figure panel. The gray arc in **(E)** indicates the megaplasmid region estimated to be lost in Δ*parAmN-1*.

To further understand the role of the Par proteins in genome maintenance, we analyzed the genome content of the *par* mutants using several methods. We inferred the relative amount of the megaplasmid by enzyme activity assays (for enzymes encoded on the megaplsmid, e.g. β-glucosidase encoded by ORF TT_P0042 and β-galactosidase encoded by TT_P0222) and we used quantitative PCR to measure the copy numbers per cell of both the megaplasmid and the chromosome. In line with the results of the growth and nucleoid visualization experiments (see above), which had indicated that *parABc* is neither involved in chromosome nor in megaplasmid bulk DNA replication and segregation, the replicon copy numbers in Δ*parABc* did not differ from those of the wild type (Figure 4A,B and C; Table 1). In strain Δ*parAmN-2*, the chromosome copy number did not differ from that of the wild type also, while the value for the megaplasmid was mildly increased as judged from the enzyme activity assays and qPCR analysis (Figure 4A,B and C; Table 1). Consistent with this, PFGE analysis also showed that the chromosome and megaplasmid were intact in this mutant (Figure 4D). In strain Δ*parAmN-1*, no carotenoid synthesis as well as no β-glucosidase or β-galactosidase activity was detectable (Figure 4A and B), indicating that these megaplasmid-encoded genes possibly were no longer present. Further qPCR, PCR and PFGE analyses showed that in strain Δ*parAmN-1*, the chromosomal DNA content appeared to be unchanged while a large portion of the megaplasmid was missing (Figure 4C,D,E and F; Table 1). The size of the resultant megaplasmid in this strain was approximately 125–130 kbp in contrast to 232.6 kbp for the wild type (Figure 4D), and the coordinates of the eliminated region could be roughly mapped (Figure 4E). It seemed that this smaller megaplasmid could not be resolved properly after replication, as duplicated, triplicated, and even quadruplicated megaplasmid sizes could be observed (Figure 4D). Because the above effects were not present in strain Δ*parAmN-2*, which differs from Δ*parAmN-1* only by the orientation of the resistance marker and by the expression levels of the truncated ParAm and ParBm proteins (see above), we conclude that inadequate amounts of ParABm in Δ*parAmN-1* led to the loss of large portions of the megaplasmid accompanied by megaplasmid resolution and segregation defects.

### Overexpression of ParAm and ParBm in *T. thermophilus*

A slight but repeatedly detectable increase of the megaplasmid copy number was observed in the Δ*parAmN-2* strain (Figure 4B and C; Table 1), which is characterized by a higher expression level of ParABm. For further clarification if the megaplasmid copy number is related to the amounts of ParAm and/or ParBm, we constructed two strains (TMP01 and TMP02) in which the *parAm* and *parBm* genes were expressed from plasmids (pMK-*parAm* and pMK-*parBm*, respectively). Both strains did not display obvious cell growth, cell morphology, cell division or DNA segregation defects. However, TMP01 and TMP02 were found to synthesize increased levels of carotenoids and displayed higher β-glucosidase activities (Figure 4A and B). Further, qPCR experiments demonstrated that both strains had 2.5 to 3.5 fold more megaplasmid copies compared to the control TMP0 strain (carrying the empty pMK18 vector), while the chromosomal copy number was unaffected (Figure 4C; Table 1). Moreover, entangled forms (catenenes) of the megaplasmids could be observed by PFGE analysis of TMP01 and TMP02, indicating that megaplasmid replication speed probably exceeded that of DNA separation and cell division (Figure 4D). Thus, it seems that both ParAm and ParBm act to promote megaplasmid replication.

### *In vivo* localization of the ParB proteins in *T. thermophilus* cells

**Figure 5:**
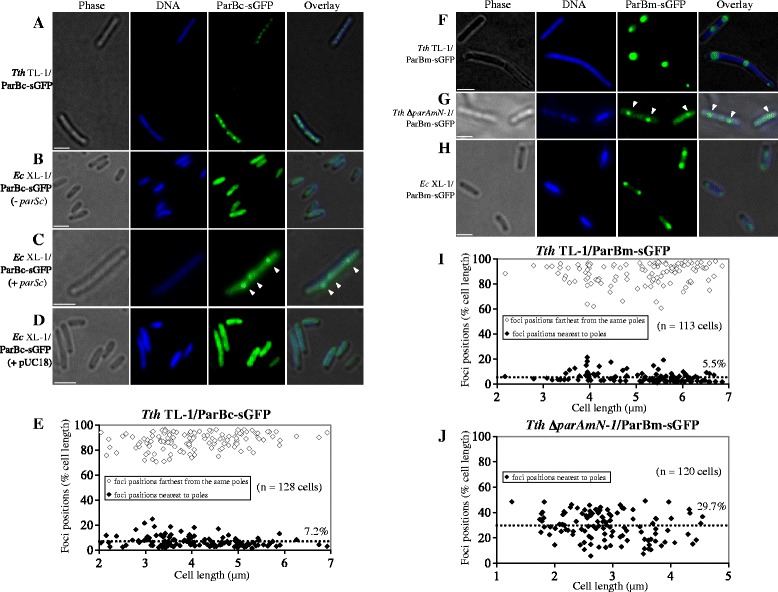
Subcellular localizations of ParBc-sGFP and ParBm-sGFP in *T. thermophilus* and *E. coli* cells. Representative cells are shown with a gallery view of phase-contrast (Phase), DNA, ParBc-sGFP or ParBm-sGFP signal, and merged images (Overlay). Scale bars, 2 μm. **(A and F)** Subcellular localization of ParBc-sGFP and ParBm-sGFP in the *T. thermophilus* TL-1 strain grown in complex medium. **(B, C, D and H)** Expression of *T. thermophilus* ParBc-sGFP or ParBm-sGFP in *E. coli* XL-1. In the absence of the *parSc* site, ParBc-sGFP was found as patches **(B)**; when *parSc* sites were provided from a plasmid, ParBc-sGFP localized as discrete foci **(C)**, and foci were not observed in the presence of the empty vector **(D)**; ParBm-sGFP formed discrete foci in *E. coli* **(H)**. **(G)** Representative image of mislocalized foci formed by ParBm-sGFP expressed in the Δ*parAmN-1* strain. **(E and I)** Relative positions of the two most pole-proximal foci of ParBc-sGFP **(E)** and ParBm-sGFP **(I)** expressed in *T. thermophilus* TL-1. Black diamonds represent the nearest-to-pole foci positions in individual cells, white diamonds represent the foci positions that are farthest from these poles. The mean position of the nearest-to-pole foci is shown with a dotted line. **(J)** The relative foci positions of 120 Δ*parAmN-1*/ParBm-sGFP cells containing one focus are shown.

Studies of ParB localization patterns in other bacteria have shown that fusions of fluorescent proteins to ParB proteins form punctate fluorescent foci representing ParB-*parS* nucleoprotein complexes in the cells [32,34,43]. To investigate the *in vivo* localization pattern of the *T. thermophilus* ParB proteins, we constructed C-terminal sGFP fusions of ParBc and ParBm. The sGFP variant used by us has been reported before [44], and it has been shown that it is able to fold and fluoresce properly when expressed in *T. thermophilus* growing at high temperatures (about 60°C). When the ParBc-sGFP and ParBm-sGFP constructs were expressed in *T. thermophilus* TL-1 (a carotenoid synthesis deficient strain isolated in our group, permitting better observation of sGFP fluorescence), well-defined fluorescent foci could be observed (Figure 5A and F) providing *in vivo* evidence that the two ParB proteins can bind *parS* sites. Obviously, the polyploid nature of the cells is unproblematic with respect to distinct foci formation. In both cases, the majority of cells contained 2–6 foci with two of them localized at the cell poles (“old” poles), and the rest localized at positions of septum formation (“future” poles) (Figure 5A and F). For better illustration, the positions of the two most pole-proximal foci were measured from the nearest poles and expressed as fractions of the cell lengths. The plot of these measurements (approximately 120 cells were randomly selected for each strain) showed that the nearest-to-pole foci of ParBc-sGFP and ParBm-sGFP were on average in only 7.2% and 5.5% distance from the cell poles, indicating ParBc and ParBm are extremely polar localized (Figure 5E and I). Assuming that the subcellular locations of ParBc and ParBm actually also mark the positions of the corresponding *parS* regions and thus the respective chromosomal and megaplasmid origin regions (see Figure 1A), it can be concluded that the origin regions of both replicons share a cellular localization near the cell poles. The fact that foci sometimes could also be detected at the cell centers or septum formation positions (“future” poles) indicated that ParB-*parS* (i.e., ParB-origin) nucleoprotein complexes might travel from cell poles to cell division positions, thus their cellular localization is dynamic.

As described above (Figure 4D), the Δ*parAmN-1* strain lacking adequate ParABm amounts displayed megaplasmid segregation defects. We tested if also the subcellular location of ParBm (i.e. the subcellular location of *parSm*) was altered in this strain by expressing the ParBm-sGFP fusion in the Δ*parAmN-1* background. ParBm-sGFP also formed discrete foci in this strain (Figure 5G). However, the foci were mostly dissociated from the cell poles (Figure 5G), i.e. most of the cells contained randomly positioned fluorescent foci. The average pole-proximal focus position (measured from the nearest poles) in cells that contained one focus was drastically increased compared with that in the wild-type cells (Figure 5J). This experiment showed that the decrease of the ParABm amounts and especially that of the ParAm amount (see Figure 2D), caused by reduced *parABm* expression, led to mislocalization of the *parSm* sites and thus of the megaplasmid origin regions.

### *In vivo* localization *of T. thermophilus* ParBc and ParBm in *E. coli* cells

In order to better understand the factors that influence the ParB proteins’ localization patterns, we expressed components of the *T. thermophilus* Par systems in *E. coli*, a host that does not encode chromosomal *parABS* system homologues. When *T. thermophilus* ParBc-sGFP was expressed in *E. coli*, the fluorescence signal was spread over the nucleoid and no foci were formed (Figure 5B). Discrete fluorescent foci, which were randomly localized in the cells, could be observed only after the *T. thermophilus parSc* site was introduced into this strain (from a plasmid pUC-Δ*parABc::kat*) (Figure 5C), and this effect was not observed in the empty vector control (pUC18) (Figure 5D). This means the ParBc subcellular localization pattern is dependent on the specific chromosomal *parS* site. On the contrary, we found that the megaplasmid ParB protein (expressed as ParBm-sGFP) formed foci when expressed alone in *E. coli* cells (Figure 5H), suggesting that there were ParBm binding sites contributed by *E.coli* or by the *parBm* coding sequence itself. Since the 14-bp palindromic sequence could be readily identified in the *parBm* gene (see Figure 1A), we favored the latter option. In summary, the different localization patterns of ParBc and ParBm in *E. coli* cells suggested that the two ParBs bind different *parS* sites.

### *In vitro* DNA binding assays of the ParB proteins to *parS* sites

**Figure 6:**
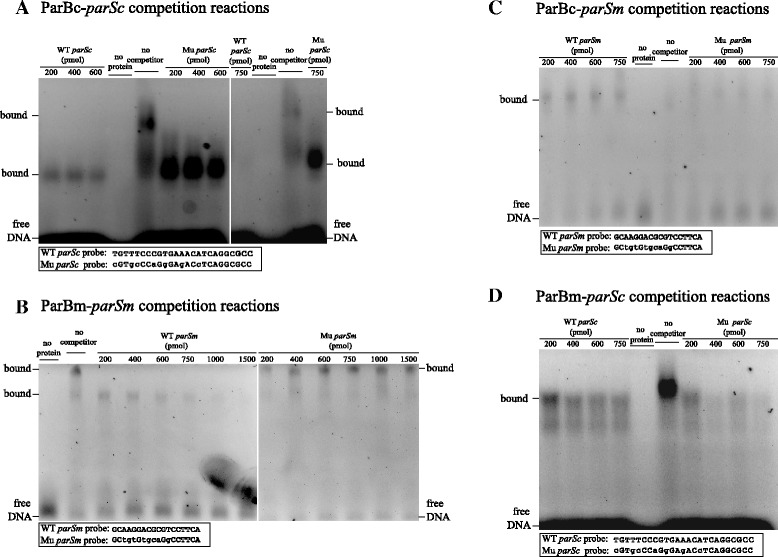
Specificity of ParB proteins binding to *parS* sites *in vitro*, tested by competition with unlabeled DNA probes in gel mobility shift assays. **(A and B)** Specificity of the ParB proteins binding to cognate *parS* sites (ParBc-*parSc* and ParBm-*parSm*) tested by the addition of unlabeled competitor DNA probes. **(C and D)** Specificity of the ParB proteins binding to non-cognate *parS* sites (ParBc-*parSm* and ParBm-*parSc*) tested by the addition of unlabeled competitor DNA probes. The 25-bp DNA probes containing the wild-type and mutant *parSc* sequences are indicated as WT *parSc* and Mu *parSc*, and the 18-bp DNA probes containing the wild-type and mutant *parSm* sequences are indicated as WT *parSm* and Mu *parSm*. All reactions contained 15 pmol FAM-labeled wild-type *parSc* or *parSm* DNA probes, and purified ParBc or ParBm proteins were added with a concentration of either 0 (no protein) or 200 pmol.

The above experiments demonstrated that the *parABc* and *parABm* systems seem to play different cellular roles, and that the two ParBs also tend to only associate with their cognate *parS* sites. To further verify these observations, *in vitro* binding of recombinant ParB proteins to the 16-bp chromosomal *parSc* site and/or the predicted 14-bp megaplasmid *parSm* site were assayed by EMSA. These assays showed that ParBc could bind *parSc* and ParBm could bind the predicted *parSm* (Additional file 3: Figure 3SA and B). Almost all DNA-binding proteins contain more than one nucleic acid binding site, and during *in vitro* DNA binding assays they possibly bind any DNA non-specifically [45]. To test binding specificity, competition experiments with unlabeled probes were performed. In both cases, the unlabeled wild-type *parSc*/*parSm* probe competed much better than the unlabeled mutant *parSc*/*parSm* probe (Figure 6A and B). Thus the 16-bp *parSc* sequence and the 14-bp *parSm* sequence were bound specifically by ParBc and ParBm, respectively. Further, we performed EMSA of the ParB proteins with their non-cognate *parS* sites. These assays showed that ParBc did not bind specifically to *parSm* and ParBm did not bind specifically to *parSc in vitro*, as the respective mutant *parS* probe competed even better than the wild-type *parS* probe (Figure 6C and D). Thus, we conclude that the two ParB proteins bind *parS* sites in a replicon-specific manner.

## Discussion

There are multiple copies of the chromosome and the megaplasmid in *T. thermophilus* [36] and whether their segregation is stringently regulated is not clear. Both the chromosome and the megaplasmid sequences of *T. thermophilus* strain HB27 revealed *par* loci. In this study, we investigate the characteristics and functions of the two *par* systems, thereby providing first insights in the mechanisms which may be involved in the genome partitioning in *T. thermophilus*.

### Characteristics of the chromosomal *par* locus

Chromosomal Par orthologs seem to possess various functions in different bacterial species, and their role in chromosome segregation is suggested to be less pivotal compared with their counterparts in plasmids. Although deletion of *spo0J* (*parB*) of *B. subtilis* leads to a considerable increase of anucleate cells during vegetative growth, the rest of the cells still exhibit a normal chromosome segregation pattern [27]; moreover, deletion of *soj* (*parA*) has no significant effect on chromosome segregation [46]. Similar observations have been made in some Gram-negative bacteria. In *Pseudomonas putida*, the *parAB* genes are not essential, and *parA* and *parB* mutations did not influence cell growth or chromosome segregation in rich medium [47]. In *V. cholerae*, deletion of *parA1* does not alter cell growth and chromosome I (chrI) partitioning; however, the polar localization pattern of the origin region is abrogated, indicating that the *parABS1* system functions to mediate the localization and segregation of the chrI origin region but not of the bulk nucleoid [32]. Our results show that in *T. thermophilus* the role of the chromosomal Par system is similar to that of the *parABS1* system of *V. cholerae*. The *parABc* null mutant generated by us did not display apparent defects with respect to cell growth rate or frequency of cells with aberrant nucleoids (Figure 3; Table 1). Further observations from experiments targeting the copy numbers of the replicons showed that the chromosomal *par* locus was probably not required for either chromosome or megaplasmid bulk DNA replication and segregation (Figure 4A,B and C; Table 1). It is likely that the *T. thermophilus* chromosomal bulk nucleoid segregation is accomplished by other mechanisms. This conclusion is in line with the view that separate and redundant mechanisms may be involved to regulate the bacterial chromosome replication and segregation [48].

However, the chromosomal *par* locus may play other roles. Indeed, the *in vitro* DNA-binding assays showed that ParBc could bind the *parSc* site specifically (Additional file 3: Figure S3A; Figure 6A), indicating it is a functional ParB protein. *In vivo*, the ParBc-*parSc* complexes localized to the poles of wild-type *T. thermophilus* cells (Figure 5A and E) and this localization was apparently dynamic, indicating that the origin regions were bound by ParBc, and the nucleoprotein complexes were driven from “old” poles to “new” poles. It has been shown that *in vitro* the *T. thermophilus* ParAc can form dimers and then associate with DNA, forming nucleoprotein filaments, suggesting that ParAc has the capacity to mediate DNA movement [41]. Thus, similar to some other Par systems [25,32], the ParBc-origin complexes could possibly be anchored to the poles via ParAc filaments. Taken together, our data indicate that *parABc* is probably involved in the chromosomal origin region localization.

### Characteristics of the megaplasmid *par* locus

The megaplasmid *par* locus is structured differently than the chromosomal *par* region. The *parABm* locus is also located in the megaplasmid origin-proximal region, and has a genetic set-up very similar to that found in some low-copy-number plasmids (Figure 1A). We could not obtain deletion mutants of the *parABm* operon or of the *parBm* gene, suggesting an essential role of *parABm*. Essentiality of *par* genes for bacterial cells has been observed for some chromosomal *par* systems. The null mutant of *parB* in *C. cresentus* is lethal [31], and direct deletion of the *parAB2* genes in *V. cholerae* chromosome II is also not feasible [34]. To our knowledge, our work shows for the first time that a (mega)plasmid *par* locus is essential for its host organism. However, a parallel can be drawn from the case of the *V. cholerae parAB2* locus on chromosome II, because this appears to be a megaplasmid-derived chromosome [49].

In the *T. thermophilus* Δ*parAmN-1* mutant, which expressed less ParABm, both the cell growth rate and the frequency of cells with irregular nucleoids were affected (Figure 3; Table 1). Furthermore, a substantial part (about 100 kbp) of the 232 kbp megaplasmid, covering the region between approximately 11 kbp and 111 kbp distance from one side of the megaplasmid origin, was lost in this mutant (Figure 4). This was not a spurious observation for just one clone, as all of 10 randomly selected Δ*parAmN-1* colonies picked up directly from the transformation plates were found to have lost the same region of the megaplasmid. These phenotypes of the Δ*parAmN-1* strain were not observed in the isogenic mutant Δ*parAmN-2* that differed from Δ*parAmN-1* only by the direction of the antibiotic resistance cassette (and thus the transcription levels of the truncated *parABm*), suggesting that they were caused by inadequate amounts of ParABm in the Δ*parAmN-1* cells. Moreover, the truncated megaplasmid in Δ*parAmN-1* seemed not to be decatenated properly, as multimeric forms of the megaplasmid could be observed by PFGE analysis (Figure 4D). In addition, the *parSm* sites (i.e. the megaplasmid origin regions) were dissociated from the cell poles and drastically mislocalized, as judged by the subcellular locations of the ParBm-sGFP fusion (Figure 5G and J). These findings suggest that *parABm* probably mediates the accurate subcellular localization and segregation of the megaplasmid, resembling the function of the Par systems in most low-copy-number plasmids and some chromosomes of other bacteria.

The observation that only part of the megaplasmid was missing is in agreement with the conclusion that *parBm* is essential in *T. thermophilus*. It is likely that not only *parBm* but also other megaplasmid regions are essential and it seems that elimination of the entire megaplasmid is lethal to *T. thermophilus*. In support of this, we were not able to cure the megaplasmid from *T. thermophilus* despite various attempts (own unpublished work). The precise reason why the megaplasmid loss is not tolerated is currently unknown.

When we overexpressed either ParAm or ParBm in wild-type *T. thermophilus* cells (TMP01 and TMP02), the megaplasmid but not the chromosomal copy numbers were increased (Figure 4A,B,C and D; Table 1). This points to a role of *parABm* in megaplasmid replication initiation and/or copy number maintenance. It is possible that the ParABm proteins can activate the factors (e.g. RepA initiator) that are involved in the megaplasmid replication. A role of the ParA and ParB proteins in genome replication has been recently proposed also for other bacteria. In *B. subtilis*, Spo0J (ParB) was found to recruit a SMC condensin protein to replication origin regions, thereby promoting chromosome segregation [50,51]; the same phenomenon was also observed in *Streptococcus pneumoniae* [52]. ParB2 encoded by the *V. cholerae* chromosome II (chrII) was also found to influence the replication of chrII, in which ParB2 appeared to promote the replication by activating RctB protein that initiates chrII replication [53]. In the chromosome of *B. subtilis* and chromosome I of *V. cholerae*, ParA was found to directly interact with the chromosome replication initiator DnaA, thereby participating in the regulation of chromosome replication [54-56]. Taken together, it is conceivable that the ParABm system in *T. thermophilus* is important for maintaining the megaplasmid through regulating its replication and segregation. Interestingly, it also seems that segregation of the *T. thermophilus* megaplasmid is coordinated with its decatenation, which is reminiscent of the situations found in the *E.coli* or *Streptomyces coelicolor* chromosomes. Mutations of the *parE* gene (encoding one of the subunits of topoisomerase IV) in *E.coli* or *S. coelicolor* lead to chromosome catenation and fragmentation, thereby affecting the chromosome segregation [57,58]. It is possible that in the *T. thermophilus* Δ*parAmN-1* cells*,* the megaplasmid could not be decatenated properly due to the inadequate ParABm amounts, thus the megaplasmid was guillotined during separation into the daughter cells, and only those cells that recombined the essential portions of the megaplasmid would then survive. Apparently, further experiments (e.g. FISH) are needed in order to define whether the irregular nucleoid cells of the Δ*parAmN-1* strain were cells that lacked the entire megaplasmid and thus were essentially dead cells or were cells that contained the chromosome and the “mini” megaplasmid.

### Chromosomal and megaplasmid Par are two independent systems

*In vitro*, the *T. thermophilus* chromosomal ParBc and the megaplasmid ParBm could bind their corresponding *parS* site in a specific manner, and the Par proteins’ binding to non-cognate *parS* sites was unspecific (Figure 6). These findings suggested that the two ParBs act only with their cognate *parS* sequences. This is supported by the *in vivo* ParB localization investigations in *E. coli* cells, as the two ParBs seemed to localize differently in this heterologous system (Figure 5B,C and H). In *E. coli* cells, we found that ParBm-sGFP could form foci, and this further confirmed the conclusion drawn from the *in vitro* ParBm-*parSm* binding experiments, that is *parBm* contains its own binding site *parSm*.

The results of the *in vitro* ParB-*parS* bindings and of the *in vivo* ParB localization experiments, together with the fact that perturbation of the expression of *parABm* only affected the replication and/or segregation of the megaplasmid but not that of the chromosome, support the hypothesis that the two Par systems function independently. The phenomenon that *parAB* function in a replicon-specific manner has also been observed in other bacteria containing more than one Par system, for example the ParAB1 and ParAB2 systems of chromosomes I and II in *V. cholerae* [59] and the Par systems of the four replicons in *B. cenocepacia* [35]. From the bacteria with multiple replicons studied so far it seems like a common theme that their Par systems behave independently of each other rather than forming a network system with shared components.

## Conclusions

One *T. thermophilus* cell contains multiple copies of the chromosome and megaplasmid. Like many bacteria, both the chromosome and the megaplasmid of *T. thermophilus* encode orthologs of the plasmid partitioning (*par*) genes, however their role in genome segregation is not known. In this study, we investigate the functions of these two Par systems in *T. thermophilus* through analysis of chromosomal and megaplasmid *par* gene mutants and ParAm/ParBm overexpression strains, as well as by using *in vitro* DNA binding assays of heterologously expressed ParB proteins and *in vivo* ParB protein localization observations. We show that in *T. thermophilus* the chromosomal ParAB system is not required for either the chromosomal or megaplasmid bulk DNA replication and segregation. It is however involved in the polar localization and separation of the chromosomal origin region. In contrast, the megaplasmid ParAB system in *T. thermophilus* probably functions to regulate the megaplasmid replication and segregation, thereby maintaining the megaplasmid. The two Par systems in *T. thermophilus* appear to function in a replicon-specific manner. Our study provides the first insights of the mode of operation of Par systems in a two-replicon, polyploid bacterium.

## Methods

### Bacterial strains and growth conditions

*Escherichia coli* XL-1 Blue (Agilent Technologies, Santa Clara, USA) was used as a host for DNA manipulations and was grown in LB medium (10 g/l tryptone, 5 g/l yeast extract, 5 g/l NaCl) at 37°C. *T. thermophilus* HB27 (DSM 7039) and its derivative strains were grown at 60°C or 70°C with vigorous shaking in rich medium (TB) or nutritionally defined medium (SH). TB medium had a pH of 7.5 and contained per litre 8 g trypticase peptone, 4 g yeast extract, and 3 g NaCl, and was prepared with a high-carbonate mineral water (Purania, DRINKPOOL GmbH, Germany). SH medium was prepared as described in [60]. The growth media were supplemented with ampicillin (100 μg/ml for *E. coli*), kanamycin (20 μg/ml for *E. coli* and *T. thermophilus*), bleomycin (“Bleocin”, Calbiochem, 15 μg/ml), chloramphenicol (12.5 μg/ml for *E. coli*), XGlc (5-bromo-4-chloro-3-indolyl-β-D-glucopyranoside, 50 μg/ml) or XGal (5-bromo-4-chloro-3-indolyl-β-D-galactopyranoside, 50 μg/ml) when appropriate. All reagents were purchased from Sigma-Aldrich (Schnelldorf, Germany) except for growth media components which were obtained from BD Biosciences (Heidelberg, Germany).

### Strains and plasmids

All strains and plasmids used are listed in Table 2. The oligonucleotides used are summarized in Additional file 4: Table S1. All allele exchange vectors (Table 2) for generating *par* mutants were derived from pUC18, and the constructs were obtained by Gibson assembly (New England Biolabs) [61]. In general, the upstream and downstream sequences (approximately 1 kbp each) of the target regions, and the gene cassette encoding thermostable resistance to kanamycin (*kat*) or bleomycin (*blm*) (chemically synthesized using sequence data from [62]) were PCR-amplified using primers that generated sufficient overlaps. The purified PCR products of the two flanking regions and the *kat*/*blm* cassette were introduced into XbaI-digested pUC18 via four-fragment Gibson assembly reactions.

**Table 2:** **Strains and plasmids used in this study**

**Name**	**Description**	**Source/reference**
**Plasmids**		
pUC18	high-copy-number cloning vector	[68]
pMK18	*E. coli*/*T. thermophilus* shuttle vector, *Tth* (*repA*), *Ec* (*oriE*), Km^R^	[63]
pUC-Δ*parABc::kat*	allele exchange vector for generating Δ*parABc*, *ori* pUC, Km^R^	this study
pUC-Δ*parABm::blm*	allele exchange vector for generating Δ*parABm*, *ori* pUC, Blm^R^	this study
pUC-Δ*parAmN-1*	allele exchange vector for generating Δ*parAmN-1*, *ori* pUC, Blm^R^	this study
pUC-Δ*parAmN-2*	allele exchange vector for generating Δ*parAmN-2*, *ori* pUC, Blm^R^	this study
pUC-Δ*parBm::blm*	allele exchange vector for generating Δ*parBm*, *ori* pUC, Blm^R^	this study
pMK-*parAm*	pMK18 derived vector, allowing overexpression of ParAm in *Tth*	this study
pMK-*parBm*	pMK18 derived vector, allowing overexpression of ParBm in *Tth*	this study
pMK-*parABm*	pMK18 derived vector, allowing overexpression of ParABm in *Tth*	this study
pET21a	expression vector, P_T7_, *lacI*, pBR322 *ori*, Amp^R^	Novagen, Germany
pET21a-*parBc*	pET21a derived vector, allowing overexpression of ParBc in *Ec*	this study
pET21a-*parBm*	pET21a derived vector, allowing overexpression of ParBm in *Ec*	this study
pMK-*sgfp*	pMK18 derived vector, allowing expression of sGFP in *Ec* and *Tth*	this study
pMK*parBc-sgfp*	pMK18 derived vector, allowing expression of parBc-sGFP in *Ec* and *Tth*	this study
pMK*parBm-sgfp*	pMK18 derived vector, allowing expression of parBm-sGFP in *Ec* and *Tth*	this study
***T. thermophilus*** **strains**	**Description**	**Source/reference**
HB27	*Thermus thermophilus*	DSM 7039
HB27Δ*bgl*	deletion of ORF TT_P0042 in HB27	[69]
TL-1	carotenoid synthesis deficient, otherwise is considered as wild type	this study
Δ*parABc*	HB27 derivative with *parABc* replaced by *kat*	this study
Δ*parAmN-1*	HB27 derivative with the N-terminal region of *parAm* replaced by *blm* in *parABm* transcription opposite direction	this study
Δ*parAmN-2*	HB27 derivative with the N-terminal region of *parAm* replaced by *blm* in *parABm* transcription co-linear direction	this study
HB27/ParABm	HB27 derivative carrying a plasmid-borne copy of *parABm*	this study
Δ*parAmN-1*/ParABm	Δ*parAmN-1* derivative carrying a plasmid-borne copy of *parABm*	this study
TMP0	HB27 derivative carrying pMK18 vector	this study
TMP01	HB27 derivative permitting overexpression of ParAm	this study
TMP02	HB27 derivative permitting overexpression of ParBm	this study
TL-1/parBc-sGFP	TL-1 derivative permitting expression of ParBc-sGFP	this study
TL-1/ParBm-sGFP	TL-1 derivative permitting expression of ParBm-sGFP	this study
Δ*parAmN-1*/ParBm-sGFP	Δ*parAmN-1* derivative permitting expression of ParBm-sGFP	this study

*Tth*, *T. thermophilus*; *Ec*, *E. coli*; Amp^R^, ampicillin resistant; Blm^R^, bleomycin resistant; Km^R^, kanamycin resistant; *Tth* (*repA*), replication origin for *Tth*; *Ec* (*oriE*), replication origin for *Ec*; *ori* pUC, replication origin for pUC18.

All the replicative vectors (Table 2) in *T. thermophilus* were derived from the *E. coli*/*T. thermophilus* shuttle vector pMK18 [63]. The constructs pMK-*parAm*, pMK-*parBm* and pMK-*parABm*, which were generated by Gibson assembly as described above, carry *parAm*, *parBm* or the entire *parABm* operon, respectively, transcriptionally fused to *kat* of pMK18. In the same manner, the plasmid pMK-*sgfp* was obtained by adding the *sgfp* coding sequence, which was chemically synthesized using sequence data from [44], to *kat* of pMK18. The constructs pMK*parBc-sgfp* and pMK*parBm-sgfp* represent translationally fused *parBc* and *parBm* to the *sgfp* gene in pMK-*sgfp*. Codons encoding four glycine residues (poly-glycine linker) were introduced between *parB* and *sgfp*, and the ParB-sGFP fusions were expressed under the same promoter of the *kat* gene in pMK18.

The plasmids pET21a-*parBc* and pET21a-*parBm* were obtained by introducing purified *parBc* and *parBm* PCR fragments into XhoI, NdeI linearized pET21a by Gibson assembly.

### Quantitative PCR

The quantitative PCR method for measuring the relative genome copies was performed as described in [64]. The chosen sites of the chromosome were near the origin (*oriCc*) and terminus (*terc*) regions, and those of the megaplasmid were the TT_P0043 locus (approximately 32 kbp from the megaplasmid origin) and TT_P0195 locus (near the megaplasmid terminus (*term*)). Standard fragments used for quantification for each chosen locus were amplified by PCR using *T. thermophilus* genomic DNA as the template. The fragments were then purified from agarose gels and photometrically quantified. A series of dilutions containing defined numbers of the standard molecules were then used as templates for qPCR to generate standard curves. Cell extracts of the strains for qPCR were prepared by harvesting defined cell numbers (determined by spectrophotometry and with a Neubauer counting chamber) from exponentially growing cultures and resuspending in cell lysis buffer (Epicentre Biotechnologies, Germany); the cell lysis efficiency was determined by cell counting. After dialysis, dilutions were prepared from the cell lysates and aliquots were used as templates for qPCR. The sizes of the target amplicons were between 100 and 200 bp, and PCR was performed using qPCR Mastermix plus with fluorescein (Eurogentec, Germany) based on the protocol provided by the manufacturer. Three independent experiments were carried out for each strain. Standard curves were constructed from the C_T_ values of the standard fragments and were later used to quantitate the genome copy numbers in the cell lysates.

### RT-qPCR

For determining the relative expression levels of the truncated *parAm* and *parBm* genes, reverse transcription-qPCR was performed. The cDNA was synthesized from total RNA samples using the Maxima First Strand cDNA Synthesis Kit (Thermo Scientific, Germany). A chromosomally located constitutively expressed gene (TT_C1610) was chosen as an endogenous reference. The relative quantification method (2^-ΔΔC^_T_) based on [65] was used in the calculations.

### Pulsed field gel electrophoresis (PFGE)

PFGE was performed as described in [66], and the CHEF-DR® III variable angle system was used for gel electrophoresis (Bio-Rad). 150 ml 1% PFGE certified agarose (Biozym Gold Agarose) prepared in 0.5 × TBE was used for gel casting. The gels were run in 0.5 × TBE for 24 h under the following conditions: 6 V/cm, 120 degree included angle, 8–50 sec switch time ramp, 14°C.

### Purification of the ParBc and ParBm proteins

ParB proteins were heterologously expressed in *E. coli* Rosetta 2 (DE3) after introduction of specifically constructed pET21a-based expression plasmids designated as pET21a-*parBc* and pET21a-*parBm*. Cultures were grown in 1 l LB medium (supplemented with chloramphenicol and ampicillin) at 37°C. When the OD_600_ reached a value between 0.7 and 0.8, protein expression was induced by the addition of IPTG at a final concentration of 1 mM and the cultures were agitated at 30°C for 4 h. The cells were harvested and lysed by sonication (UP200S, Hilscher, Teltow, Germany). After sonication, the crude cell extracts were centrifuged at 4°C at 15,000 *g* for 30 min and the supernatants were subjected to affinity purification using Protino Ni-IDA 2000 columns (Macherey Nagel, Germany).

### Electrophoretic mobility shift assay (EMSA)

For DNA binding assays, a 25-bp or a 18-bp DNA fragment that contained the chromosomal or megaplasmid *parS* sequence was used as the probe. The probes were 6-carboxyfluorescein (FAM)-labeled and were generated by hybridization of two complementary oligonucleotides. The chromosomal *parS* probe had the sequence:

5′-TGTTTCCCGTGAAACATCAGGCGCC-3′(WT *parSc*), and the megaplasmid *parS* probe had the sequence: 5′-GCAAGGACGCGTCCTTCA-3′ (WT *parSm*). The binding reactions (25 μl) were performed in 50 mM KCl, 10 mM Tris–HCl (pH 7.0), 1 mM EDTA, 1 mM DTT, 4% glycerol, 0.02 μg/μl Poly (dI-dC), and contained 15 pmol of FAM-labeled probe and varying amounts of ParB proteins. The reactions were incubated at 25°C for 30 min and then applied on 1% agarose gels prepared in 1 × TBE buffer. The ParB-*parS* binding competition experiments were performed using both the unlabeled probes containing the wild-type *parSc* or the wild-type *parSm* sequence (wild-type competitor), and unlabeled probes that contained seven base-pair and eight base-pair changes in the *parSc* and *parSm* sites respectively (mutant competitor). The mutant *parSc* probe had the sequence: 5′-cGTgcCCaGgGAgACcTCAGGCGCC-3′ (Mu *parSc*), and the mutant *parSm* probe had the sequence: 5′-GCtgtGtgcaGgCCTTCA-3′ (Mu *parSm*). They were also generated by hybridization of two complementary oligonucleotides.

### Fluorescence microscopy

For fluorescence microscopy, the cells from liquid cultures were collected by centrifugation (5000 *g*, 10 min), washed once with 1 × PBS buffer and resuspended in the same volume of 1 × PBS buffer. Staining was performed by the addition of DAPI (4′,6-diamidino-2-phenylindole-dihydrochloride) with a final concentration of 0.2 μg/ml, and if necessary, by the addition of 10 μg/ml 6-carboxyfluorescein (CFS), followed by incubation at RT for 20 min. The residual dyes were washed off and the cells were resuspended in 1 × PBS buffer. Fluorescence microscopy was performed with a Zeiss Axio-Imager M1 microscope using filter sets “DAPI” for DAPI, “AF488” for CFS and for the sGFP fluorescence of strains that expressing ParB-sGFP, respectively. The micrographic images were taken with an AxioCam MRm camera and analyzed with the Image J (NIH, USA) and AxioVision software (Carl Zeiss, Germany).

### β-glucosidase activity assay for *T. thermophilus*

β-glucosidase activity was measured with exponentially growing cells as described in [67]. The enzyme assays were performed with three independently grown cultures.

## Additional files

Additional file 1: Figure S1. Predictions of the chromosomal and megaplasmid origin and terminus regions in *T. thermophilus* HB27. The GenSkew software (http://genskew.csb.univie.ac.at/) was used to compute the normal and cumulative GC skew for the chromosome and the megaplasmid. The windowsize and stepsize for the chromosomal sequence were both set to 1000 bp, and those for the megaplasmid sequence were both set to 100 bp. (A) Cumulative GC skew of the chromosomal sequence. The maximum indicating the chromosomal terminus position is at 558, 731 bp, the minimum representing the chromosomal origin region position is at 1, 524, 671 bp. (B) Cumulative GC skew of the megaplasmid sequence. The maximum and minimum values are at the megaplasmid sequence positions 189, 545 bp and 71, 689 bp, indicating the positions of the megaplasmid terminus and origin regions, respectively. Additional file 2: Figure S2. Genotype confirmation of the *parABm* and *parBm* mutants in *T. thermophilus*. (A) Genotype confirmation of the *parABm* mutants by PCR using genomic DNA as template and primers flanking the deleted region (primer pairs parm-F/parm-R). The *in silico* predicted sizes are 3.99 kbp for the wild-type allele and 2.73 kbp for the Δ*parABm* allele. (B) Genotype confirmation of the *parBm* mutants by PCR (primer pairs parm-F/parm-R-2). The predicted sizes for the PCR products are 3.39 kbp for the wild type and 3.13 kbp for the Δ*parBm* allele. Additional file 3: Figure S3.
*In vitro* DNA binding of ParBc to *parSc*, and of ParBm to *parSm* measured by gel mobility shift assays. All reactions were performed under the same condition as described in the Methods section. Shifted DNA species were labeled with “bound”, free DNA species were labeled with “free DNA”. (A) Gel shift assays were performed with 15 pmol FAM-labeled DNA probe containing the 16-bp *parSc* site (probe sequence: 5′-TGTTTCCCGTGAAACATCAGGCGCC-3′), and with various concentrations of ParBc. (B) Gel shift assays were performed with 15 pmol FAM-labeled DNA probe containing the predicted 14-bp *parSm* site (probe sequence: 5′- GCAAGGACGCGTCCTTCA-3′) and with various concentrations of ParBm. Additional file 4: Table S1. Primers used in this study.
