# Supplementary material for: Characterization of chromosomal and megaplasmid partitioning loci in Thermus thermophilus HB27
Source: BMC Genomics. 2015 Apr 18;16(1):317. doi: 10.1186/s12864-015-1523-3 (PMC4409726; doi:10.1186/s12864-015-1523-3)

Additional file 2: Figure S1. Predictions of the chromosomal and megaplasmid origin and terminus regions in *T. thermophilus*.

A GC-skew plot for the chromosomal sequence

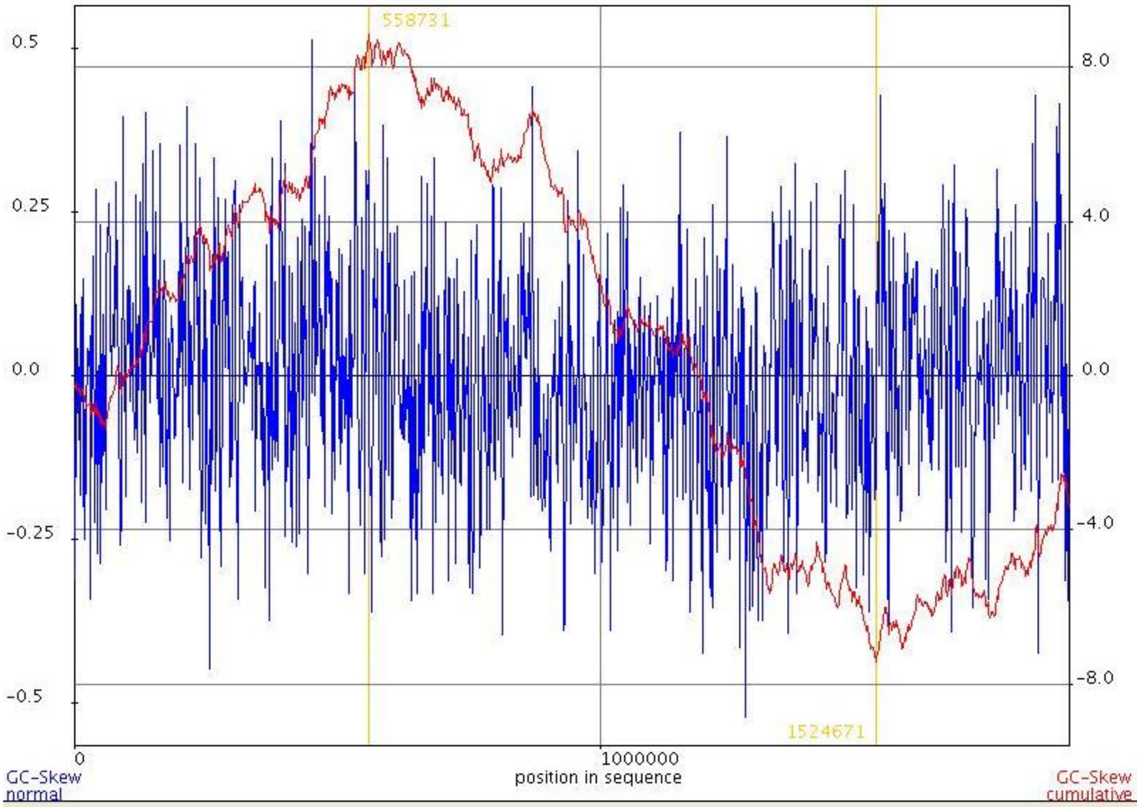

B GC-skew plot for the megaplasmid sequence

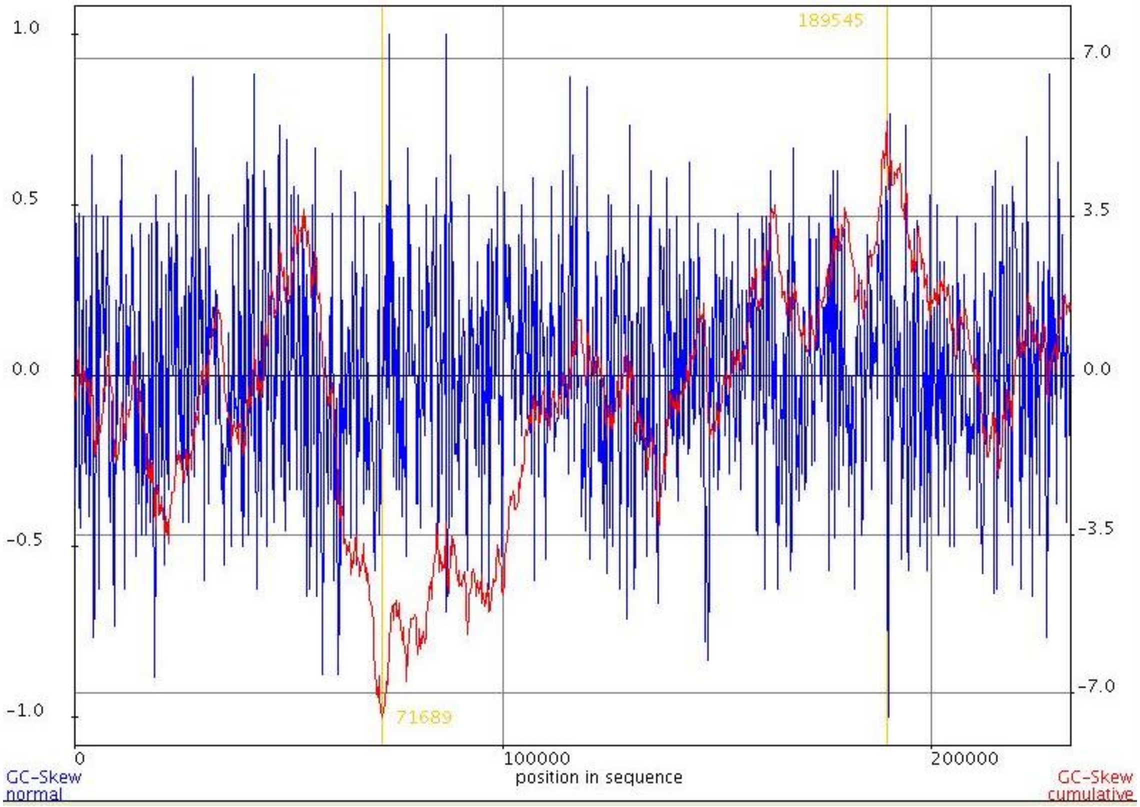

Supplement: Additional file 1: Figure S1. — Predictions of the chromosomal and megaplasmid origin and terminus regions in T. thermophilus HB27. The GenSkew software (http://genskew.csb.univie.ac.at/) was used to compute the normal and cumulative GC skew for the chromosome and the megaplasmid. The windowsize and stepsize for the chromosomal sequence were both set to 1000 bp, and those for the megaplasmid sequence were both set to 100 bp. (A) Cumulative GC skew of the chromosomal sequence. The maximum indicating the chromosomal terminus position is at 558, 731 bp, the minimum representing the chromosomal origin region position is at 1, 524, 671 bp. (B) Cumulative GC skew of the megaplasmid sequence. The maximum and minimum values are at the megaplasmid sequence positions 189, 545 bp and 71, 689 bp, indicating the positions of the megaplasmid terminus and origin regions, respectively. [file 12864_2015_1523_MOESM1_ESM.pdf]
