# Supplementary material for: Characterization of chromosomal and megaplasmid partitioning loci in Thermus thermophilus HB27
Source: BMC Genomics. 2015 Apr 18;16(1):317. doi: 10.1186/s12864-015-1523-3 (PMC4409726; doi:10.1186/s12864-015-1523-3)

Additional file 3: Figure S2. Genotype confirmation of the *parABm* and *parBm* mutants in *T. thermophilus*.

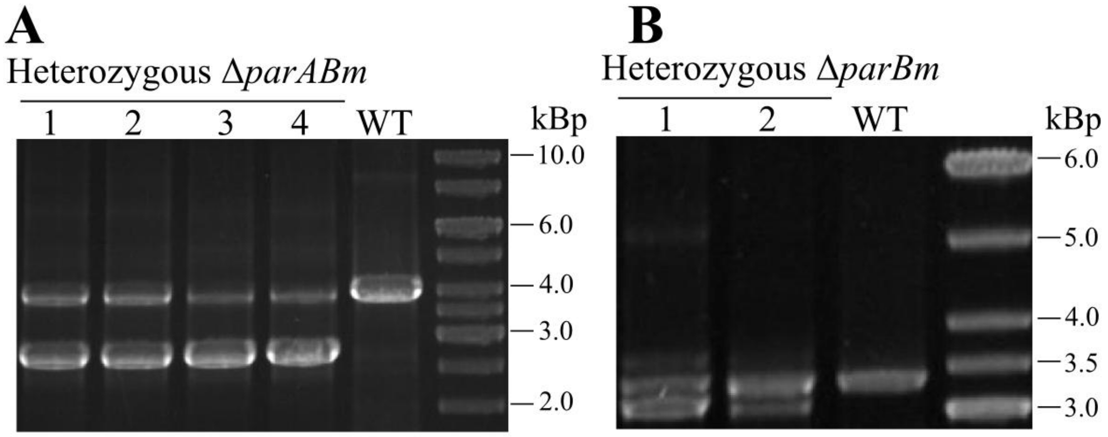

Supplement: Additional file 2: Figure S2. — Genotype confirmation of the parABm and parBm mutants in T. thermophilus. (A) Genotype confirmation of the parABm mutants by PCR using genomic DNA as template and primers flanking the deleted region (primer pairs parm-F/parm-R). The in silico predicted sizes are 3.99 kbp for the wild-type allele and 2.73 kbp for the ΔparABm allele. (B) Genotype confirmation of the parBm mutants by PCR (primer pairs parm-F/parm-R-2). The predicted sizes for the PCR products are 3.39 kbp for the wild type and 3.13 kbp for the ΔparBm allele. [file 12864_2015_1523_MOESM2_ESM.pdf]
