# Supplementary material for: Characterization of chromosomal and megaplasmid partitioning loci in Thermus thermophilus HB27
Source: BMC Genomics. 2015 Apr 18;16(1):317. doi: 10.1186/s12864-015-1523-3 (PMC4409726; doi:10.1186/s12864-015-1523-3)

Additional file 4: Figure S3. *In vitro* DNA binding of purified ParB proteins to their cognate *parS* sites by gel mobility shift assays.

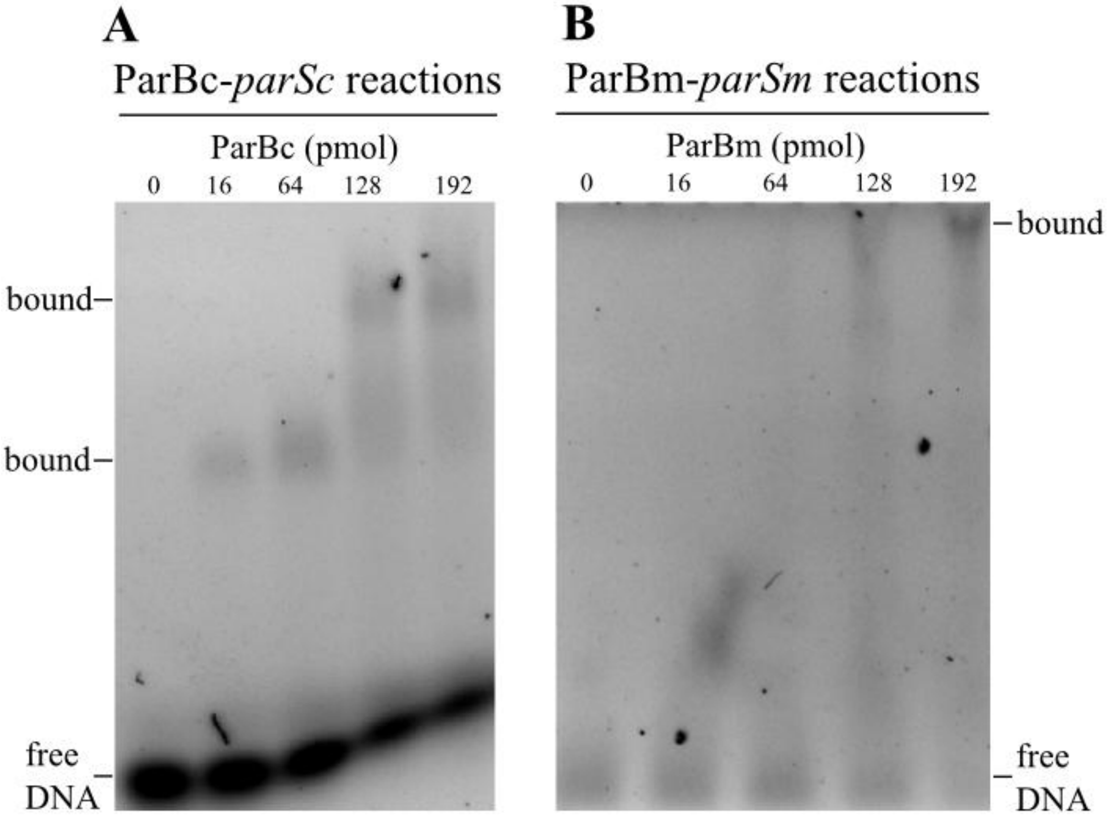

Supplement: Additional file 3: Figure S3. — In vitro DNA binding of ParBc to parSc, and of ParBm to parSm measured by gel mobility shift assays. All reactions were performed under the same condition as described in the Methods section. Shifted DNA species were labeled with “bound”, free DNA species were labeled with “free DNA”. (A) Gel shift assays were performed with 15 pmol FAM-labeled DNA probe containing the 16-bp parSc site (probe sequence: 5′-TGTTTCCCGTGAAACATCAGGCGCC-3′), and with various concentrations of ParBc. (B) Gel shift assays were performed with 15 pmol FAM-labeled DNA probe containing the predicted 14-bp parSm site (probe sequence: 5′- GCAAGGACGCGTCCTTCA-3′) and with various concentrations of ParBm. [file 12864_2015_1523_MOESM3_ESM.pdf]
