# Supplementary material for: Characterization of chromosomal and megaplasmid partitioning loci in Thermus thermophilus HB27
Source: BMC Genomics. 2015 Apr 18;16(1):317. doi: 10.1186/s12864-015-1523-3 (PMC4409726; doi:10.1186/s12864-015-1523-3)
Supplement: Additional file 4: Table S1. — Primers used in this study. [file 12864_2015_1523_MOESM4_ESM.pdf]

Additional file 1: Table S1. Primers used in this study.

Name	Sequence (5'-3')	Usage
parABc-1-F	catgcctgcaggtcgactCCTCGGCTTCCTCAAGCTCTTC	amplify the *parABc* flanking region 1 for pUC-Δ*parABc::kat*
parABc-1-R	agagcgcccaatacgcaaaccgGAAGGGCAAGGTGGTGATCCAG	
parABc-2-F	cttggaggagaaacgccGGCCCTTAGCATAACGGATACC	amplify the *parABc* flanking region 2 for pUC-Δ*parABc::kat*
parABc-1-R	cggtacccggggatcctCAAGTACGCGGGCTACATTG	
kat-1-F	cggtttgcgtattgggcgctctTCCCCGGGAGTATAACAGAAACC	amplify *kat* for pUC-Δ*parABc::kat*
kat-1-R	ggcgtttctcctccaagAATTCCGTTCAAAATGGTATG	
parc-3	gcataccattttgaacggaaCCGAAGAGGACGCGCACCGC	together with parABc-1-F, amplify the template for the probe of Southern blot detection of Δ*parABc* in *Tth*
parABm-1-F	tgcatgcctgcaggtcgactGAACATCCACGGTGCCGTAG	amplify the *parABm* flanking region 1 for pUC-Δ*parABm::blm*
parABm-1-R	gttatactcccggggatcccGTCTTGCGGAAGGAGAAGGC	
parABm-2-F	gactgatctagaggatccccATCTCGCTCACGGGAACCAG	amplify the *parABm* flanking region 2 for pUC-Δ*parABm::blm*
parABm-2-R	gctcggtacccggggatcctACGAGACCGGGAAGTACGAG	
blm-1-F	gccttctccttccgcaagacGGGATCCCCGGGAGTATAAC	amplify *blm* for pUC-Δ*parABm::blm*
blm-1-R	ctggttcccgtgagcgagatGGGGATCCTCTAGATCAGTC	
parBm-1-R	gttatactcccggggatcccTAGAGGGGCCGAGGTAACG	together with parABm-1-F, amplify the *parBm* flanking region 1 for pUC-Δ*parBm::blm*
parBm-2-F	gactgatctagaggatccccGTGCCCAGAACCTCGTCCAG	amplify the *parBm* flanking region 2 for pUC-Δ*parBm::blm*
parBm-2-R	ggtacccggggatcctctagCTACTGGTACGTGCGGGAAC	
blm-4-F	GGGATCCCCGGGAGTATAAC	amplify *blm* for pUC-Δ*parBm::blm*
blm-4-R	GGGGATCCTCTAGATCAGTC	
parm-F	AGTCAAGGCCACGGGTGTCTTC	PCR detection of the Δ*parABm* mutants
parm-R	CCAGACCATCGTCTACGTCTTC	
parm-R-2	CCAGGTTTCGCCCTCCACA	together with parm-F, detect the Δ*parBm* mutants
parAmN-1-F	tgcatgcctgcaggtcgactCTTCTTGCCCTTGAGGATCG	amplify the *parAmN* downstream flanking region for pUC-Δ*parAmN-1* and pUC-Δ*parAmN-2*
parAmN-1-R	gttatactcccggggatcccTACGTGCGGGAACGGGAGGG	
parAmN-2-F	gactgatctagaggatccccATGGGCCTAGACTATCCCAA	together with parABm-2-R, amplify the *parAmN* upstream flanking region for pUC-Δ*parAmN-1* and pUC-Δ*parAmN-2*; together with parABm-2-R, amplify the template for Southern blot detection of the *parAmN* mutants
blm-2-F	ccctcccgttcccgcacgtaGGGATCCCCGGGAGTATAAC	amplify *blm* for pUC-Δ*parAmN-1*
blm-2-R	ttgggatagtctaggcccatGGGGATCCTCTAGATCAGTC	
blm-3-F	gggatccccgggagtataacGGGGATCCTCTAGATCAGTC	amplify *blm* for pUC-Δ*parAmN-2*
blm-3-R	ggggatcctctagatcagtcGGGATCCCCGGGAGTATAAC	

Table S1 continued		
pMK-1-F	GGATGTGCTGCAAGGCGATTAAGTTGG	amplify the PMK18 backbone for pMK-*parAm*, pMK-*parBm* and pMK-*parABm*
pMK-1-R	TCAAAATGGTATGCGTTTTG	
parAm-F	caaaacgcataccattttgaTGGGGGATACTTGGCAAACG	amplify *parAm* for PMK-*parAm*; parAm-F combining with parBm-1-R, amplify *parABm* for pMK-*parABm*
parAm-R	aatcgccttgcagcacatCCTCGTCCAGCCGGCTCATTCC	
parBm-1-F	caaaacgcataccattttgaCTGGAGGAGGTGGCGGAATG	amplify *parBm* for PMK-*parBm*; parBm-1-R combining with parAm-F, amplify *parABm* for pMK-*parABm*
parBm-1-R	aatcgccttgcagcacatccAACGAGAGGGCGTTACCTCG	
parAmRT-F	TGCAGGAACTCCTCCGTCAG	RT-qPCR measuring the truncated ParAm expression levels in Δ*parAmN-1* and Δ*parAmN-2*
parAmRT-R	ACAACCGGGTGCTGGAGAAG	
parBmRT-F	CACCTCTTCCACCGACTTCC	RT-qPCR measuring the ParBm expression levels in Δ*parAmN-1* and Δ*parAmN-2*
parBmRT-R	TCCTGGACCTCTCCGAGAAG	
P43-F	ACCTGCGCCTTGTCCATGTC	generate TT_P0043 standard fragment for qPCR
P43-R	TGGGCGCAGGCCACATAAAC	
P43-R-1	AGGCCATCTCCGAGGGAAAG	together with P43-F, use for qPCR detection of TT_P0043 copy number
terCm-F	TCCTGGTCCAGTGAAGACAAG	generate TT_P0195 (*terCm*) standard fragment for qPCR
terCm-R	GGCAGTACTCCGTGTTTGAAG	
terCm-F-1	TTGCATAAGGTGGCCTTCG	use for qPCR detection of TT_P0195 (*terCm*) copy number
terCm-R-1	GTCTTGGCGGTGTACTTCTTG	
oriCc-F	TCAAGGAGAAGGGCTACAG	generate *oriCc* standard fragment for qPCR
oriCc-R	CCTTGTAGCTCACGGAAAC	
oriCc-F-1	ACGCCATCCTGGTCAAGGTG	use for qPCR detection of *oriCc* copy number; amplify the reference gene for measuring ParABm expression levels in Δ*parAmN-1* and Δ*parAmN-2*
oriCc-R-1	AGGTCGGCGATGAAGCTGTC	
terCc-F	CCGGCAGGTAGACGTCAAAG	generate *terCc* standard fragment for qPCR
terCc-R	TGAGCCGGAGGGAGTTTGAG	
tercC-F-1	AGGTGACCACCACGCTTTCG	use for qPCR detection of *terCc* copy number
tercC-R-1	TTAGGCCGCCAGGATCAGTACG	
1-F	CGCCTGGAGAACGTCTTGTG	the 1 to 10 primer pairs for detecting megaplasmid sequence loss in the Δ*parAmN-1* mutant
1-R	GCTCTTTCGCCGACAACGTG	
2-F	CGTAGAGGAGGAGCATCAC	
2-R	TCCGGGAGAAGGTCTACTG	
3-F	CTTCGGCCTCTACTACGTG	
3-R	GCCTCTTCCAGAAGGTCTC	
4-F	AGGCTTAGGCACCACAAC	
4-R	TTGCCCAGGTGGCTATTCAG	
5-F	GCTCCTCTACCACCTGTCTG	

**Table S1 continued**		
5-R	CTCTTTCACCTCCGCCTACTC	
6-F	CCTCCTGTGGCTTTCTATC	
6-R	GCTCTGGAGAGGAGTTTG	
7-F	CACCATCCAGCGCAGAAAGC	
7-R	ACGACTTCCGGCCCGATTAC	
8-F	AGGCGTAGGGAAGGTTGTC	
8-R	AGGCGTAGATGGGAGGAAG	
9-F	CCGCACAGTATCTCGGTCTC	
9-R	CAGGAAGCGCCCTCTTAAGC	
10-F	TGGACACCGATCAGGTAAC	
10-R	GGTGGGTATGCGATTCAAG	
parBc-F	tttaagaaggagatatacataTGTCCAGGAAGCCTAGCGGT	amplify *parBc* for pET21a-*parBc*
parBc-R	gtggtggtggtggtggtgctcgagCGCCTGGTAGCCGAGGCGCC	
parBm-2-F	tttaagaaggagatatacatATGAGCCGGCTGGACGAGGT	amplify *parBm* for pET21a-*parBm*
parBm-2-R	tcagtggtggtggtggtggtgctcgagCCTCGGCCCCTCTAGGACCC	
pMK-2-R	ttgcgcatatgcctcacacctcctTCAAAATGGTATGCGTTTTG	together with pMK-1-F amplify pMK18 backbone for pMK-*sgfp*
sgfp-F	gtgtcaaaacgcataccattttgaAGGAGGTGTGAGGCATATGC	amplify *sgfp* for pMK-*sgfp*
Sgfp-R	aatcgccttgcagcacatccTCTTACTTGTAGAGCTCGTC	
pMKfp-F	ggaggaggaggaCGCAAGGGCGAGGAGCTCTT	amplify the pMK-*sgfp* backbone for pMK*parBc-sgfp* and pMK*parBm-sgfp*
pMKfp-R	TCAAAATGGTATGCGTTTTG	
parBcfp-F	caaaacgcataccattttgaGAGGAGGTGATGGCCCGTGT	amplify *parBc* for pMK*parBc-sgfp*
parBcfp-R	cccttgcgtcctcctcctccCGCCTGGTAGCCGAGGCGC	
parBmfp-F	caaaacgcataccattttgaCTGGAGGAGGTGGCGGAATG	amplify *parBm* for pMK*parBm-sgfp*
parBmfp-R	cccttgcgtcctcctcctccCCTCGGCCCCTCTAGGACCC	

Sequences that create overlaps for the Gibson assembly reactions are in lowercase.

Tth, *T. thermophilus*.
